# L1 retrotransposon expression in circulating tumor cells

**DOI:** 10.1371/journal.pone.0171466

**Published:** 2017-02-06

**Authors:** Ioannis Papasotiriou, Katerina Pantopikou, Panagiotis Apostolou

**Affiliations:** Research Genetic Cancer Centre S.A., Industrial Area, Florina, Greece; The Ohio State University, UNITED STATES

## Abstract

Long interspersed nuclear element 1 (LINE-1 or L1) belongs to the non-long terminal repeat (non-LTR) retrotransposon family, which has been implicated in carcinogenesis and disease progression. Circulating tumor cells (CTCs) are also known to be involved in cancer progression. The present study aimed to compare the L1 expression between circulating tumor cells and non-cancerous samples. Blood samples were collected from 10 healthy individuals and 22 patients with different types of cancer. The whole blood cells were isolated using enrichment protocols and the DNA and RNA were extracted. RT-qPCR was performed for L1-ORF1 (open reading frame 1) and L1-ORF2, using 18S rRNA as the reference gene. The data were analyzed with the Livak method and statistical analyses were carried out with the Mann-Whitney and Kruskal-Wallis tests. In parallel with the above molecular biology experiments, FISH experiments were performed on the interphase nuclei of the cells for the detection of ORF2 RNA. DNA analysis revealed the presence of both ORF1 and ORF2 in all samples. RNA expression experiments demonstrated that ORF1 was not expressed in all samples, while ORF2 was expressed at varying levels in the non-cancer samples and the samples representing the different cancer types. A significant difference in ORF2 expression was observed between the CTCs and non-cancer samples (p = 0,00043), and significant differences were also observed between normal and lung (p = 0,034), pancreatic (p = 0,022), prostate (p = 0,014), and unknown primary of origin (p = 0,0039) cancer samples. Cytogenetic analysis revealed higher levels of ORF2 in the nuclei of CTCs than in normal samples. This study highlights the significant difference in L1-ORF2 expression between CTCs and normal samples. The increased expression levels observed for CTCs may be correlated with the characteristic features of these cells.

## Introduction

Long interspersed nuclear element-1 (LINE-1 or L1) is the most abundant and only autonomously active member of the non-LTR retrotransposon family, which comprises approximately 17% of the human genome. L1 expression is more abundant than was initially expected, and therefore a source of significant interindividual variation [1]. The retrotransposons not only contribute to human genome evolution but are also implicated in oncogenesis [2]. Recent experimental data indicated that L1 ORF2 affect specific transcription factors implicated in stemness pathway like NANOG, OCT3/4 and SOX2 or other genes involved in Epithelial to Mesenchymal transition [3].

Circulating tumor cells (CTCs) are cells that have detached from the primary tumor and enter the blood or lymphatic stream, thereby causing a secondary tumor [4]. CTCs may also include cancer stem cells (CSCs) as a subset population [5]; this group of cells is therefore very important and useful for analyzing the relationship between retrotransposition and oncogenesis.

The current study first examined the presence and expression of L1 across a wide spectrum of circulating tumor cells derived from different types of cancer as well as from healthy individuals. We then explored any correlations in expression between normal cells and cancer cells, as well as among the normal samples and each type of cancer. Finally, cytogenetic assays were used to study L1 ORF2 expression at the cellular level.

## Methods and materials

### Sample collection

Blood samples of 20 mL each were collected from 10 healthy individuals and 22 patients in sterile 50 mL tubes (4440100; Orange Scientific, Belgium) containing 7 mL 0,02 M EDTA (E0511.0250; Duchefa Biochemie B.V., The Netherlands) as an anti-coagulant. Healthy individuals were identified as healthy or with non-malignant disease by their physicians. For the cancer samples, the following cancer types were included: breast cancer, one patient with stage I, one patient with stage II, one patient with stage III and five patients with a non-applicable stage; prostate cancer, three patients with a non-applicable stage; pancreatic cancer, three patients with a non-applicable stage; lung cancer, three patients with a non-applicable stage; and finally, five patients with unknown primary of origin cancer. The samples were incubated at room temperature on a roller for 30 min and then sent to the laboratory for analysis. The transit time of the samples from collection point to the laboratory did not exceed 72 h. The study was performed between January and June 2016.

### Sample preparation

Whole blood cells were centrifuged with 4 ml of polysucrose solution (Biocoll separating solution 1077; Biochrom, UK) at 1600rpm for 20min. Mononuclear cells, lymphocytes, platelets, and granulocytes were collected after centrifugation and washed with phosphate-buffered saline (PBS, P3813; Sigma-Aldrich, Germany). Cells were incubated for 10 min in lysis buffer comprising 154 mM NH_4_Cl (31107; Sigma-Aldrich), 10 mM KHCO_3_ (4854; Merck, Germany), and 0,1 mM EDTA in deionized water, to lyse the erythrocytes. The samples were then centrifuge-washed with PBS. Cells from the healthy donor were then incubated at 4°C for 30 min with CD45 magnetic beads (39-CD45-250; Gentaur, Belgium), while those from cancer patients were incubated with CD326(EpCAM) microbeads (130-061-101; Miltenyi Biotec) at 4°C for 30 min. Following incubation, the samples were placed in a magnetic field, collected, and then washed with PBS. The CD45-negative selected cells (non-cancerous) and the EpCAM-positive cells (cancerous) were isolated and cultured in 12-well plates (4430400N; Orange Scientific) with RPMI-1640 and 10% FBS until they reached appropriate confluence for further experiments (qPCR). CTCs were validated with PCR reactions including specific markers (CK19, EpCAM) or markers to exclude other types of cells (CD31, N-cadherin).

### Molecular analysis

DNA from the blood samples and RNA from the cell cultures were extracted using the MagCore Genomic DNA Whole Blood Kit (MGB400-02; RBC Bioscience, Taiwan) and MagCore Total RNA Cultured Cells Kit (MRC-02; RBC Bioscience), respectively. RNA was removed from the DNA isolates with RNase A (19101; Qiagen, Germany) and DNA was removed from the RNA isolates with the NucleoSpin rDNase Set (740963; Macherey-Nagel, Germany). The purified DNA and RNA were evaluated spectrophotometrically. Then, 1 μg of each RNA sample was used as the template for cDNA synthesis with the PrimeScript RT Reagent Kit (RR037A; Takara Bio, Japan). Real-time quantitative polymerase chain reactions (RT-qPCR) were then performed with the Maxima SYBR Green Supermix (K02222; Fermentas, UK) on both the cDNA and DNA samples. Specific primers for each marker and for an endogenous control gene (18S rRNA) were designed with Genamics Expression 1.1 software (Genamics™, New Zealand) (Table 1). Primer sequences were evaluated with BLAST to exclude those that would amplify undesired genes [6]. The PCR program was as follows: initial denaturation at 95°C for 10 min, 40 cycles of denaturation at 95°C for 10 s followed by annealing at 60°C for 30 s. Melting curve analysis was followed from 65°C to 95°C with 0,5°C increments of 5 s in each step. The qPCR products were electrophoresed on an agarose gel and melting curve analysis was performed to validate the results. Data were analyzed according to the method described by Livak and Schmittgen [7].

**Table 1:** Primer sequences.

Gene	Forward Primer (5’–3’)	Reverse Primer (5’–3’)
18S rRNA	TGCCCTATCAACTTTCGATGGTAGTC	TTGGATGTGGTAGCCGTTTCTCA
L1-ORF1	AGAACGCCACAAAGATACTCCTCG	CTCTCTTCTGGCTTGTAGGGTTTCTG
L1-ORF2	AAACTGAACAACCTGCTCCTGAATG	CTACACACTGCTTTGAATGCGTCC
L1-ORF2 (Probe)	AAACCCATCTCATGTGCAGAGACA	TTCTGTGGGATCGGTGGTGATA

Sequences of primers used in molecular and cytogenetic assays.

### Cytogenetic assays

#### Sample preparation

Peripheral blood mononuclear cells (PBMCs) from healthy donors were resuspended in medium containing RPMI-1640 (R6504; Sigma-Aldrich), 1% L-glutamine (G5792; Sigma-Aldrich), 20% FBS (FB-1001/500; Biosera, US), 1% penicillin/streptomycin (P0781; Sigma-Aldrich) and 2% phytohemagglutinin PHA (M5030; Biochrom), and cultured for 72 h. EpCAM positive cells from cancer patients were resuspended in medium containing RPMI-1640 (R6504; Sigma-Aldrich) and 10% FBS (FB-1001/500; Biosera). The cells from patients were cultured in RPMI-1640 medium (R6504; Sigma-Aldrich) until they reached 80% confluence. Subsequently, the cultures were supplemented with 0,05 μg/mL colcemid and incubated for a further 2 h to arrest the cells in metaphase. The cells were washed with PBS, trypsinized with trypsin solution 0,25% (T4049; Sigma-Aldrich) and incubated for 5 min to detach the cells. The trypsin was inactivated with an equal amount of media containing 10% FBS. The cells were then centrifuged at 1600 rpm for 5 min, resuspended in 7 mL KCl and incubated for 18 min at 37°C.

After incubation the cells were centrifuged at 1600 rpm for 5 min and resuspended with 5 mL Carnoy’s fixative. This step was repeated at least twice until the pellets were visibly white and swollen. The pellets were resuspended in fixative and 10 μL of each suspension was dropped onto a dry, clean slide from a height of approximately 1 cm. The slides were dried overnight at room temperature.

#### Labeling

The L1 probe was produced with endpoint PCR using fluorescent dUTPs (NU-803-FAMX-L; Jena Bioscience, Germany). The probe was validated with spectrophotometry and agarose gel electrophoresis. The primer length was 1093 bp.

#### FISH

Following the overnight drying step, the slides were incubated in 2× saline-sodium citrate (SSC) buffer (S6639; Sigma-Aldrich) and 50% formamide (476711LF; Sigma-Aldrich) for 20 min at 60°C and allowed to cool to room temperature. The hybridization cocktail comprised 0.7 U RNase (19101; Qiagen), L1 probe at a final concentration of 5 ng/μL, hybridization buffer (6J6701; Abbott Molecular, US), and molecular biology-grade water (95284; Sigma-Aldrich), and 7 μl was added to each slide. Individual slides were covered with a 22×22 mm coverslip sealed with rubber cement. The slides were then denatured at 92°C for 2,5 min and hybridized at 40°C overnight in ThermoBrite (Abbott Molecular) or in a humidified chamber. The next day the slides were incubated in 2× SSC buffer containing 0.1% Tween 20 (1610781; Bio-Rad Laboratories, Inc., Italy) at 60°C for 15 min, followed by incubation in the same buffer for 10 min at room temperature, with a final incubation in 0.2× SSC for 10 min at room temperature. For the observation of interphase nuclei with a fluorescence microscope, antifade DAPI (06J50-001; Abbott Molecular) was used. The photographs were captured with a 100× objective and analysis was carried out with CytoVision software version 4.5.2 (Leica Biosystems, Germany). There were counted twenty cells from different parts of the slide to ensure random selection without introducing a bias.

### Statistical analyses

The qPCR results were tested for normality with the Kolmogorov-Smirnov test. Mann-Whitney and Kruskal-Wallis tests were also performed on the qPCR data to test for significant differences between the various samples. A significant p value was defined as <0.05. The statistical analyses were performed with PAST version 2.10 [8].

### Ethics approval

This study was not a clinical trial and did not include any interventions in the patients. All procedures were conducted according to the standards of Safety, Bioethics and Validation [9]. The study was reviewed and approved by the Bioethical Committee of the Research Genetic Cancer Centre Group (S1 Fig). All patients provided written consent for the use of their sample in the present study. The patients retained the right to withdraw their sample until the date when the sample was received at the laboratory and tested.

## Results

### Molecular assays

**Fig 1:**
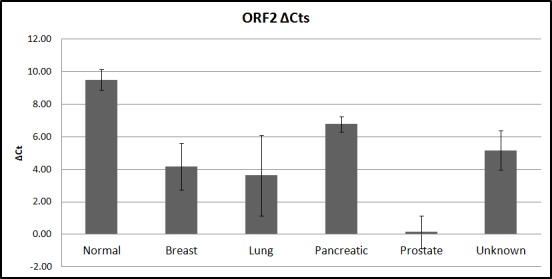
Analysis of L1-ORF2 expression. Comparative qPCR analysis of L1-ORF2 expression in normal and different cancer samples. 18S rRNA served as the reference gene for normalization. A lower ΔC_t_ indicates higher gene expression. The error bars represent the standard error of mean.

DNA analysis revealed that all samples were positive for the presence of ORF1 and ORF2. The RNA qPCR experiments demonstrated that L1-ORF1 was not expressed in all samples, while ORF2 was. The ORF2 expression levels were significantly different between the normal samples and the cancer samples (p = 0,0004; Fig 1). Significant differences were also observed between normal and lung (p = 0,03), normal and pancreatic (p = 0,02), normal and prostate (p = 0,01) and normal and unknown origin (p = 0,004) cancer samples. The normal and breast CTC analysis did not indicated a difference in expression (p = 0,06). Regarding the differences between the various cancer types, only prostate and unknown primary origin cancer samples exhibited a significant difference (p = 0,03) (S1 File).

**Fig 2:**
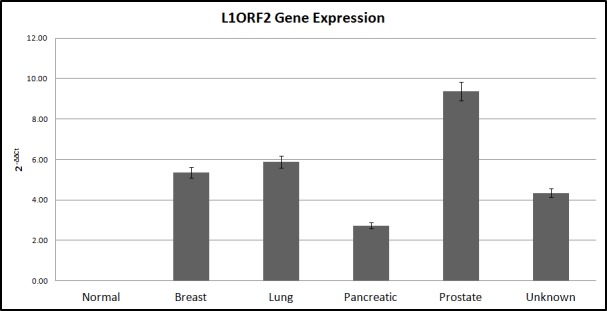
Normalized L1-ORF2 expression in different cancer types. Comparative qPCR data of L1 ORF2 expression across different cancer types, where the expression levels in the cancer samples were normalized with the mean expression in healthy samples. Samples were normalized to non-cancer samples with the Livak method and 18S rRNA was used as the reference gene.

The Livak method revealed that relative to the normal samples, ORF2 exhibited the highest expression in prostate CTCs, followed by breast and lung CTCs (Fig 2). The expression level was lower for unknown origin of primary tumor samples and the lowest expression was observed for the pancreatic cancer samples.

### Cytogenetic assays

**Fig 3:**
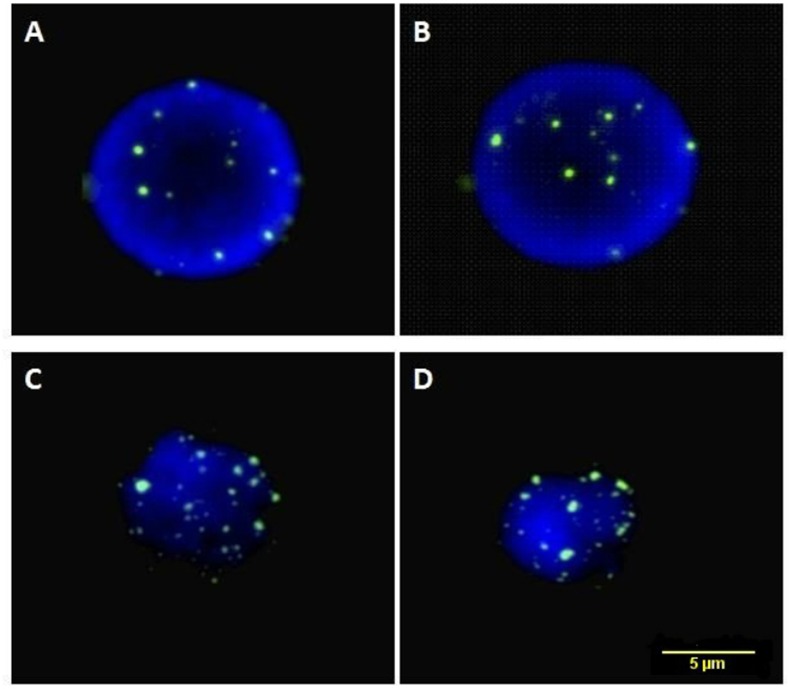
FISH in the interphase nuclei of normal and lung CTCs. A–B: Nuclei (blue) from non-cancerous samples hybridized with a probe for L1-ORF2 expression (green). C–D: Nuclei from CTCs derived from lung cancer patients.

FISH experiments on the interphase nuclei revealed that in CTCs the level of ORF2 signal (18,50 ± 0,91 counts per nucleus) was significantly higher compared with non-cancer cells (11,1 ± 0,74; p = 0,001) (Fig 3, S1 File).

## Discussion

The L1 retrotransposon encodes two open reading frames. ORF1 is responsible for chaperone activity, while ORF2 has endonuclease and reverse transcriptase activity [10]. According to literature and experimental data, L1 has been implicated in carcinogenesis via chromatin remodeling at the epigenetic level [11], chromosomal rearrangements [12] and double-strand break formations in the DNA [13]. Another experimental study demonstrated that L1 can be inserted in the APC tumor suppressor gene, which may be correlated with initiation of colorectal cancer[14]. Furthermore, L1 insertions have been observed in the CADPS2 gene in human embryonic stem cells [15]. L1 methylation status has proven very important in embryonic development, since demethylation at the preimplantation stages is correlated with mitotic errors in chromosome segregation [16]. According to Garcia-Perez et al. [17], embryonic cells have the ability to silence genes that are delivered by L1 retrotransposition, thus maintaining an undifferentiated state. In breast cancer cells, L1 reverse transcriptase activity is essential for maintaining stemness, since inhibition thereof promotes differentiation [18].

First and foremost, the present study demonstrated that while there was no ORF1 gene expression in the different cancer types tested, expression of the ORF2 gene differed among the different types of cancer and was statistically significant between cancer and non-cancer samples. The above results support the hypothesis that L1 retrotransposition may contribute to tumorigenesis. The review of Sciamanna et al [19] proposes a model for cell transformation and tumorigenesis driven by LINE-1-ORF2 reverse transcriptase activity at an epigenetic level. Furthermore, similarly enhanced expression of ORF2 has been observed in colon and prostate cancer [20]. The role of L1 in CTCs is still unclear, and very little data on tumor microvesicles are available. Based on these experimental data, tumor microvesicles contain retrotransposon elements that are responsible for gene transfer [21]. Therefore, the role of ORF2 appears to mainly lie in retrotransposition, while ORF1 may play a secondary role that is not directly correlated with tumorigenesis. Among the different types of cancer we tested in this study, it is noteworthy that prostate-derived CTCs exhibited the highest expression followed by breast cancer, indicating that L1 may be affected by or may affect various hormones. However, the study of El-Maari et al. [22] demonstrated that only gender and not the age or natural hormone cycles affect the methylation status of L1.

The methodology of FISH can complement data obtained from whole genome sequencing, inverse PCR or other methods currently used for studies of L1 [23]. The FISH method enables the quantitation of ORF2 signals at the single-nucleus level. However it remains unclear where L1 prefers to insert and whether a targeting mechanism exists to direct these insertions within the genome. Last but not least, the FISH experiments supported the data obtained in the molecular biology-based assays, as the data demonstrated that the ORF2 signals in the CTC nuclei were significantly higher than in the normal samples.

## Conclusion

In summary, it is clear that L1-ORF2 RNA expression differs between normal and cancer cells. The CTCs, which are implicated in metastasis and cancer progression, have higher expression of this gene than healthy individuals. Therefore, ORF2 could potentially be used as a biomarker or prognostic tool, or it could potentially serve as a novel druggable target. Further experiments should be performed across a range of cancer types to develop these concepts further.

## Supporting information

S1 Fig Ethical Approval of the present study. (JPG)

S1 File Raw Data of PCR and FISH Experiments. (XLSX)
