# Supplementary figures and images for: L1 retrotransposon expression in circulating tumor cells
Source: PLoS One. 2017 Feb 6;12(2):e0171466. doi: 10.1371/journal.pone.0171466 (PMC5293242; doi:10.1371/journal.pone.0171466)

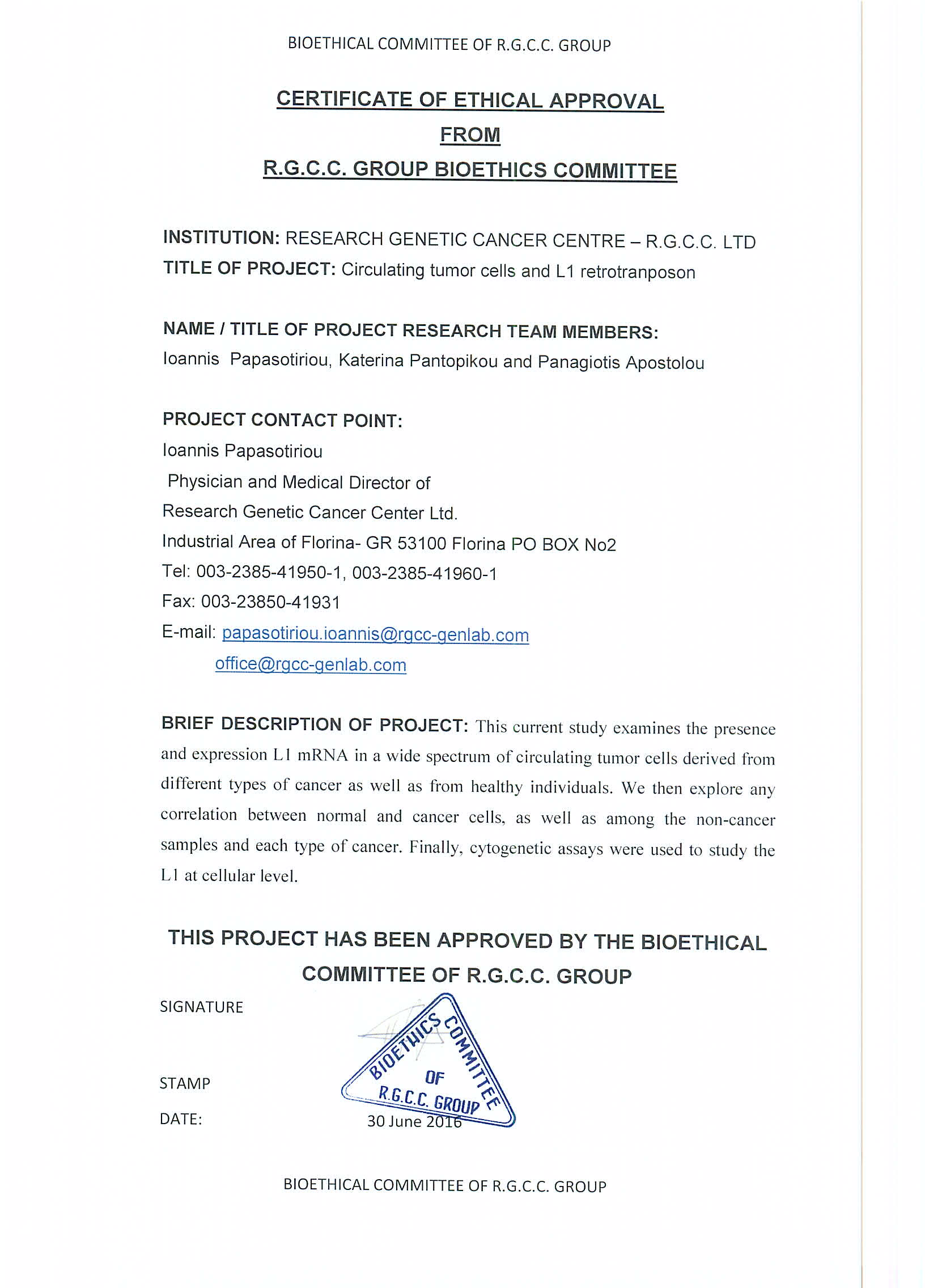

Supplement: S1 Fig — (JPG) [file pone.0171466.s001.jpg]
